# Clinical Lectures on the Diseases of Women and Children

**Published:** 1855-09

**Authors:** 


### BIBLIOGRAPHICAL NOTICES.

*Clinical Lectures on the Diseases of Women and Children.* By GUNNING S. BEDFORD, A. M., M. D., Professor of Obstetrics, the Diseases of Women and Children, and Clinical Midwifery in the University of New York. New York: 1855.

This work consists of a series of Clinical Lectures, or rather, "running commentaries upon disease," delivered from the obstetric chair of the University of New York, and originally reported by several medical gentlemen for the American Lancet, in which journal most of them have been published. The objects and scope of the book are told in the following paragraph concerning the establishment of an Obstetric Clinic :

"It was with the hope of affording you an opportunity of studying the various maladies of the female practically, and also the diseases of children, that I embarked in the enterprise of establishing an *Obstetric Clinique*, which would enable me to bring before you the most interesting of these affections, and discuss in detail their nature, causes, symptoms, complications and treatment." (Page 8.)

In their present collected form, these Lectures constitute a handsome 8vo. volume of 563 pages, a sort of Clinical Hand-Book of Obstetricy, in which the student and the junior members of the profession generally, will find much that is interesting and practically instructive.

"If you will take a retrospect of the last few weeks, you will find, on recurring to your note books, that numerous cases of every day occurrence, the very character of cases which you are most in need of, because they constitute the every day work of professional life, have been brought before you. Their causes, symptoms, diagnosis, pathology, complications and treatment have been fully discussed. These cases, after being prescribed for, have returned, and you have been the witnesses as to the result of the treatment; you have seen whether our views have been sound and worthy of thought, or whether they have been speculative, and, like most hypothetical doctrines, apocryphal, and, therefore, unsafe as guides in the practice of the healing art." (Page 73.)

The typography and general appearance are excellent. With regard to the title—*Obstetric Clinique*—we would simply remark that consistency demands that it be written, in one of two ways, either *Clinique Obstetrique*, or Obstetric Clinic.

The eminently practical ideas of the author, clothed in simple and perspicuous language, are delivered in quite an attractive, affable and off-hand manner. We regret to perceive, however, that this pleasant style is marred by a certain occasional colloquiality, which, notwithstanding the prefatory disclaimer of Prof. B., cannot but be regarded as objectionable by the judicious and time-valuing reader. This very feature, which of late has been obtruding itself somewhat too much in our medical literature, renders it difficult to assign to the work under consideration its true literary and scientific rank. For so far as it is colloquial, it is popularized, and therefore loses that strictly professional character which every work emanating from an authoritative source should possess. Moreover, professional books, whose construction is such as to attract both the popular and the scientific eye, seldom succeed in acquiring permanence. They form a sort of hybrid literature, which, like its prototypes in nature, the hybrid races of animals and men, ultimately die out and leave no mark. But a still more serious objection is implied in the quaint expression of an old English writer, "*ye bookes be ye roades unto knowledge.*" The shorter these "roades" the better; the more clear, simple and concise the language of a writer, the more rapidly and satisfactorily do we acquire his ideas. Time is thus saved, and time is momently becoming of more value as the demand upon its use increases. With the widely-spread, and in many instances illy-digested literature of a comprehensive profession before him, the student cannot afford to lose the little time at his disposal in searching through many pages for a single item of positive knowledge, which, when found, may oftentimes, like Gratiano's reasons, be not worth the search. The active practitioner, whose busy carriage allows him no rest through the long day, "in silence and at night" endeavors to meet and grapple with the responsible and arduous duties of his vocation, by seeking that knowledge, which to him is indeed power. Such an one turns instinctively to the author who, in the fewest words, gives him the best and most reliable information. The lofty thoughts of England's great poet find their expression in brief and simple terms. Who does not know that this is the secret of Shakespeare's greatness. We speak advisedly on this subject, for in so doing, we utter the sentiments of many whose daily path lies through and among books, and who are often compelled to exclaim, that "big books are great evils." Certainly verbosity is highly reprehensible in a scientific treatise.

These "Clinical Lectures," 30 in number, and comprehending the details of over 200 cases of disease, may be regarded as so many disconnected or isolated chapters upon the theory and practice of obstetric medicine. Their want of connection or relationship is manifestly due to the author's desire of rendering the volume what it no doubt is, a faithful representative of his clinic as it occurred before the class of the University of New York.

In the first Lecture, the reader will find some excellent remarks on organic and functional diseases of the uterus; their rank and importance in the nosological scale; the crude ideas entertained with regard to them by the ancients, even by Hippocrates and his school; and the comparatively recent origin of our knowledge concerning their pathology and therapeutical indications. The circumstances which modify these affections, and the causes of their apparent infrequency in former times, are also discussed briefly and to the point. In this latter connection the author makes some very practical and judicious observations upon female hygiene. The latter part of this lecture is taken up with the examination and treatment of the following cases of disease, viz: profuse menstruation from debility; acute external otitis; gonorrhœal ophthalmia; suppression of the menses from cold; and infantile pertussis.

An examination of the cases composing any one of these lectures is sufficient to show the disadvantageous character of the clinical system of teaching as at present in vogue in our medical schools. In one or two hours, at the most, numerous cases of disease, differing from each other in the most marked manner, are passed in rapid review before an audience of young men, the majority of whom have assembled for the first time, perhaps, to look disease in the face and study its protean history. This panoramic method of displaying disease is by no means calculated to assist the student in the acquisition of clear and philosophical notions concerning etiology, diagnosis, therapeutics and the like. In the effort to make the collegiate clinic varied and attractive, accuracy and extent of knowledge are too often sacrificed to mere superficial examinations, which latter must ultimately lead to routineism and all its attendant evils. We are of the opinion that these disadvantages could easily be obviated by occupying the clinical hour with a series of closely allied diseases; or better still, with one particular disease accompanied by cases illustrative of its various modifications.

Examples of chlorosis, muco-purulent vaginal discharge, pruritus pudendi, amenorrhœa, the effects of undue lactation, and scarlatinous albuminuria, make up the second Lecture. The remarks upon chlorosis are every way judicious, and such as every practical physician must concur in. It is an extremely interesting affection, and one which deserves close attention and study on account of the obscurity of its origin and primary cause. The few hasty observations at the close of the lecture upon the connection between albuminous urine on the one hand, and arrest of the cutaneous function, followed by renal congestion on the other, are as just in philosophy as they are valuable in practice.

Among the cases treated of in the third Lecture, the remarks on uterine hydatids and concealed pregnancy particularly recommend themselves to the practical physician. The reader, we are sure, will peruse with interest the graphic description of a case of fibrous tumor simulating pregnancy; not only on account of its surgical and obstetrical importance, but also on account of the important and delicate interests often involved in such cases. The narration of such a case constitutes one chapter at least in the unwritten but daily and anxiously acted history of medical experience and responsibility. Such cases show in a clear light the status which the physician occupies in society, and the extent and grandeur of his relations to his fellow creatures.

In Lecture fourth, we have a dissertation upon vaginal discharges, intestinal worms, ulcerative carcinoma of the cervix uteri, &c., with a brief exposé of human credulity and the system of quackery as that hydra-headed monster appears in New York.

Our limits forbid any other than a very hasty glance at the contents of the remaining lectures of the "Obstetric Clinique."

The 5th, 6th, 7th and 8th lectures present nothing of especial interest, except, perhaps, some remarks on the causes, symptoms, diagnosis and treatment of ulcerations of the cervix uteri. In Lecture IX. the subject of uterine diseases is continued. Near the close of it, will be found a case of suppressio mensium following an attack of scurvy. In Lecture X. the most interesting cases are two of steatomatous and sarcomatous tumors, both containing hair. In the 12th Lecture, among a variety of other matter, Dr. B., takes occasion to combat the general custom of applying two ligatures to the umbilical cord. He makes use of but one, because, 1st, "the small quantity of blood which flows from the untied extremity of the cord, consists merely of the disgorgement of the vessels on the fœtal portion of the placenta, and does not come directly from the system of the mother. 2d,

This very disgorgement, in my opinion, assists in the more prompt expulsion of the after-birth." (p. 200.) Lecture XIII. presents us with some cases of menstrual disease, fibrous tumor of the uterus and ovarian tumors, with observations on the diagnosis of the latter, and the use of the uterine sound. The reader will also find here reported a case of incipient paralysis of the inferior extremities, dependant upon defective menstruation. Lecture XIV. introduces us to the physical and moral changes which the female economy undergoes at the epoch of puberty; several pages are devoted to the time of commencement of the menstrual flux, its source, causes, symptoms and periodicity. In the 15th Lecture, the obstetric surgeon, the medical jurist and the philanthropist will alike find food for reflection. A detailed account is here given of two cases of imperforate os tincæ, accompanied with retention of the menses; this retention producing so much abdominal swelling in one, as to be mistaken for pregnancy. In both patients the os was perforated by means of a curved trocar, and egress given to about two quarts of menstrual blood, with the effect of affording speedy relief from suffering. Then follows the history of a "case of imperforate os tincæ in a pregnant woman, the result of injuries to the neck of the uterus from repeated attempts at abortion, and on whom was performed the operation of vaginal-hysterotomy, with safety to both mother and child." The increasing frequency of criminal attempts at abortion, the undisguised boldness and utter recklessness of life with which these attempts are often pursued, and the necessarily dangerous and at times fatal effects which follow, are evidenced in a case detailed by Dr. B., for which we refer our readers to page 255.

An example of procidentia vesicæ will be found in Lecture XVI. Lecture XVII. opens with some general observations upon the diseases of infancy, their importance and fatality, and the anatomical and physiological peculiarities of the infant. Passing over the next two we find in Lecture XX. a case of trismus nascentium, a disease whose pathology is still very obscure, notwithstanding the investigations of Clark, Holland, Sims and others. With regard to the treatment of this affection we may here observe, that in two very well marked cases we have witnessed decided benefit from the combined use of belladonna and iodide of potassium. Lectures XXI. to XXV. inclusive, contain the details of some fifty cases, which though of almost daily occurrence, present features of more or less interest. Lectures XXVI., XXVII. and XXVIII. are mainly taken up with observations on infantile hygiene, therapeutics, &c. Lecture XXIX. we regard as the most interesting of the entire series. It is in reality a short essay upon uræmia, one of the most important varieties of toxæmia or blood poisoning, and one, moreover, which amply illustrates the indebtedness of therapeutics and practical medicine to the untiring and philosophical investigations of the physiological chemist. Much attention has recently been directed to this subject in consequence of the doubts created by the experiments of Frerichs and others, concerning the poisonous nature of urea. Although this substance has been known as a healthy ingredient of the urine for nearly 100 years, and although it has been carefully studied by many skilful chemists, yet its origin and its influence upon the economy, especially the nervous centres, when retained in unusual quantities in the mass of blood, are still mooted points. The opinion has very generally prevailed that the accumulation of urea in the blood is sufficient to produce coma, convulsions and their attendant train of nervous phenomena. This view has been combatted from different experimental stand-points with much success. Segalas, Tiedemann, Mitscherlich, Gmelin, Cl. Bernard, Barreswill, Stannius, Frerichs and others have shown that the kidneys of living animals may be extirpated with but little subsequent disturbance of the nervous system. Again, Courten, Bichat, Gaspard, Vauquelin and others have repeatedly injected urea and urine into the veins, and in every instance failed to observe any convulsive movements. Lastly, Bright, Christison, Rees and Frerichs have in several instances detected large quantities of urea in the blood of man, without the slightest symptom of uræmic toxication. Both Vauquelin and Segalas have suggested the administration of urea as a diuretic. On the other hand, Frerichs claims to have demonstrated that in every case of uræmia a change of urea into the carbonate of ammonia takes place; and that the symptoms which characterize this disease can all be produced by the injection of carbonate of ammonia into the blood. In addition to his own numerous experiments, he finds considerable support for these views in the experiments and observations of Orfila, Bernard and Barreswill, Lehmann, Christison, Jakchs, &c.

An interesting fact in this connection is the relation of albuminuria to uræmia. From the investigations of Robin, Tegart, Franz Simon and others, it appears that the presence of albumen in the urine is not necessarily followed by uræmia. Robin suggests that in the oxygenation of the albumen of the blood we are to look for the origin of urea. He thinks that where this oxygenation is to any extent interfered with, albuminuria sooner or later appears. But, the fact that albumen in the urine is sometimes accompanied by an increase of urea in the blood, militates strongly against this opinion. The origin of urea has long been a vexed question in chemico-physiological science. Lehmann, Frerichs, Bidder and Schmidt and their adherents, maintain that this compound is derived from the assimilated histoplastic or albuminous food on the one hand, and from the necessary disintegration and absorption of albuminous tissue during fasting on the other. It will be seen that the former is the interrupted, and the latter the continuous element in this double source. Liebig, Bischoff and others of that school contend that we are to seek for the formation of urea in the metamorphosis of the nitrogenized tissues alone.

The cause and semeiologic value of albumen in the urine are also matters of great interest and importance to the practical physician as well as to the student of physiology. Our author very properly objects to the view of Dr. Williams who considers albuminuria as the result of renal congestion alone. This pathological condition we have every reason to attribute to a highly complex cause. Peculiar alterations in the composition of the blood; organic and physiological changes in the kidney itself; compression of the renal veins, by the gravid uterus, tumors, &c., are all capable of producing albuminous urine.

After a few general remarks upon the nature of the secretory process, its importance as the grand eliminating agent, by which the blood is maintained in its normal condition, Dr. B. writes. We may suppose,

"That in most of the diseases which we have enumerated as being occasionally accompanied with **albuminuria**, such, for example, as cholera, scarlatina, diabetes, &c., the constituents of the blood become changed by the introduction either of a poison or some other unusual substance. If this occur, it is quite manifest that the blood is no longer normal, and because of its altered condition, its elaboration in the kidney will also be modified. In other words, in lieu of the ordinary elements contained in the urine, we shall sometimes find albumen, an absence of urea, etc."

"Again, the blood is changed in pregnancy, various circumstances tending to this modification, viz., the formation of kiestine, the secretion of milk, the quantity of blood materials passing through the circulation of the fœtus, and the diseases of the embryo itself, not to speak of its excretions, some of which we know enter the blood of the mother. These, then, being so many influences capable of altering the constituents of the blood, will they not account, in some instances, for the occasional presence of albuminuria in the pregnant female?"

"I have no experiments to offer with the view of demonstrating that the menstrual blood positively contains noxious materials, but I argue the affirmative of this question from certain pathological states, which we observe to follow an abnormal condition of the catamenial function. For instance, in one hundred unmarried women who may labor under suppression of the menses from the operation of any of the influences known to produce this result, such as cold, mental emotion, etc., we will discover that in at least ninety-five the suppression will be followed by more or less disturbance of the nervous system. In some, it is true, the symptoms will be light and evanescent, but in others they will assume a more marked character, sometimes even producing mania, and at others coma, epilepsy, catalepsy, chorea, etc. May not these phenomena be due to a species of toxæmia traceable to the poison of the menstrual blood upon the nervous centers?

"This opinion seems to be confirmed by the important fact that all the nervous disturbances cease with the return of the function. I have enjoyed full opportunities for observing the effects on the system of the various forms of menstrual aberration; and I have also noticed an extremely interesting and significant circumstance—a circumstance which certainly tends to corroborate the hypothesis that the derangements of function are owing to a species of blood-poisoning. The circumstance to which I allude is this: when the catamenial discharge becomes suddenly or otherwise abnormally arrested, the urinary secretion is usually diminished in proportion to the intensity of the nervous symptoms—and what is still more significant is, that the nervous disturbance will yield in proportion to the effects of diuretic and sudorific remedies. There is no error as to the fact—its truth is readily susceptible of demonstration." (pp. 525-6-7.)

Of all the causes which operate in the production of albuminuria, obstruction of the renal circulation is perhaps the most common. The experiments and observations of Robinson, Meyer, Frerichs, Lee, Velpeau, Stokes, Cruveilhier, and many others, show conclusively that obliteration of renal veins, whether by ligature or otherwise, is almost invariably followed by albuminous urine. "In gestation, and especially primiparæ, albuminuria is often caused by the pressure of the impregnated uterus on the renal vessels. In 106 multiparæ, Blot has found eleven women whose urine contained albumen, while in 99 primiparæ, thirty exhibited albuminuria. The proportions, therefore, for the former are as 1 to 10—the latter as 1 to 3." This difference is probably due to the fact that in first gestations the abdominal parietes being firmer and more resisting, the gravid uterus is pressed backwards against the blood-vessels with more force, while in multiparæ the walls of the abdomen are so relaxed as to allow the womb to gravitate forwards and thus prevent this pressure and subsequent congestion of the renal capillaries.

In the treatment of uræmia, a knowledge of the conditions influencing the amount of urea in the economy is obviously of the highest importance to the physician. The recent valuable contributions to this branch of urological science—the results mainly of the many careful and elaborate investigations of the German chemists—are worthy of very close attention, since without these guides it is impossible to institute a rational treatment of this disease. The amount of urea excreted from the system is influenced by the quantity of water used as drink, the kind and amount of food, accessory diet, certain inorganic matters taken in with the ingesta, exercise, medicine, certain diseased conditions of the body, &c. According to Bischoff, excessive water-drinking gives rise to a more rapid elimination of urea by the kidneys, in consequence of its increased disintegrating effects upon the nitrogenized tissues. The experiments of Böcker confirm this view, while those of Schmidt lead to a directly opposite result. Seigmund and Bischoff have both experimented upon the influence exerted by the quantity and quality of the food upon the metamorphosis of the nitrogenous tissues, and through this latter upon the amount of urea excreted in a certain time. They found that the formation and excretion of urea in a given time was directly related to the amount of azotized food digested in that time; and that the consumption of gelatinous matters increased, while the use of fats diminished the excretion of urea. The combined experiments of Heller, Böcker and Lehmann, show very conclusively that the free use of tea, coffee, alcohol, &c., and those substances which are classed together, by systematic writers, under the head of "the accessory diet," is attended with diminished urea in the urine; while chloride of sodium, according to Bischoff, decidedly increases the quantity of urea excreted. Active exercise, wasting disease, many of the phlegmasia, as pneumonia, pleuritis, meningitis, acute tuberculosis, endocarditis, &c., all augment the formation and elimination of urea; while the chronic neuroses and most renal diseases diminish this compound. Attention is also beginning to be directed to the influence of medicinal agents upon the salts of the urine; and although the laborers in this field of research are but few in numbers, yet they have accomplished sufficient to justify very hopeful anticipations of the ultimate results. We can but allude to a few of the experiments upon the action of certain diuretic medicines. According to Prof. Krahmer, of Halle, the administration of colchicum and guaiacum, decidedly increases the amount of urea excreted in the urine. Böcker has quite recently shown that similar results follow the use of strongly concentrated phosphoric acid; while phosphate of soda produces a reverse effect. Laveran and Millon assert that Rochelle salt, in small doses, increases the amount of urea and diminishes that of uric acid. From his experiments upon rabbits, Siegmund has shown that the amount of urea is augmented by cubebs and the ethereal extract of cantharides. Lastly, Dr. Parkes claims the same effects for potash.

On page 534 of the volume before us, the reader will find the following conclusions, which, in the present state of physiological science, we have reasons for considering as fairly deducible from the above facts.

"1st. Uræmia is a nervous disturbance arising from a peculiar blood-poisoning.

"2d. Albuminuria is often connected with uræmia, but is not the cause of it.

"3d. Disease of the kidney will often produce albuminuria, but in a great many cases albuminuria exists without true disease of the gland, and as a consequence of an active, or a merely passive congestion, and it will also result from a variety of nervous disturbances.

"4th. If urea be a poison, the quantity of it which accumulates in the blood in cases of extirpation of the kidneys in animals, or in suppression of urine in man, is not sufficient to produce any manifest deleterious effect.

"5th. According to Frerichs, uræmia is merely a poisoning by the carbonate of ammonia, which is a product from the decomposition of urea.

"6th. The treatment of uræmia must consist in the free use of diuretics, sudorifics and purgatives, the most suitable diuretics for this purpose being colchicum and guaiacum."

The prominent topic in the last lecture (XXX.) is sterility, its causes, treatment, &c.

We have thus, in a hasty manner, laid before our readers a summary of the contents of the "obstetric clinique." One additional comment remains to be made. In numerous places throughout the work, the author shows very truthfully that physiology is a prominent and indispensable auxiliary in the formation of a rational, and therefore philosophical system, of therapeutics. And in most instances he is happily accurate in his application of the facts of physiological science to the details of the diagnosis and treatment of disease. Occasional errors in this respect, however, will be detected by the careful reader, errors which are perhaps justly attributable to the clinical system itself. For very certain it is that every part of the human fabric is so closely connected with all the rest, and all the functions, organic and animal, are so continuously interwoven with each other, that when the finger of disease touches an organ, a thorough and most scrutinizing examination is required in the majority of cases to separate the positive from the sympathetic symptoms, to avoid confounding effects with causes, and lastly to point out the *fons et origo mali.* But this thoroughness of examination, this slow and laborious mode, the only one truly successful, of arriving at a positive diagnosis, demands more time than the clinical hour can afford. Hence the temptation, at once to avoid severe mental labor and save time, is often too great to be resisted. Thus errors are introduced into the logic of medicine which might otherwise have been avoided.

By referring to page 11 of the volume under notice, the reader will find the following words:

"The case of the patient before us, presents an example of profuse menstruation purely from debility, and is the result of an atonic state of the system and more especially of the uterine vessels, which together with the increased fluidity of the blood from the loss of its fibrin, will at once account for this particular form of hemorrhage."

Now, Andral and Scherer have shown, as the results of their observations, that the proportion of fibrin in the blood actually increases instead of diminishes in consequence of venesection, even when the latter process is pushed to the extreme, as in severe cases of pneumonia. The analyses of Franz Simon indicate that in the blood of horses undergoing starvation, the fibrin increases from 3 to 4 parts—the normal mean—to 11 or 12 per 1000 parts. The same phenomenon is manifest in anæmic and chlorotic cases, and also in healthy individuals during the fatigue induced by violent exercise, when the tissues undergo a more rapid disintegration and metamorphosis. The debility of this case is perfectly compatible with the reduction of the red corpuscles, which we know to constitute the potential elements of the blood, and which are rapidly abstracted by blood-letting, hemorrhage, etc. That the fluidity of the blood was one element in the cause of this profuse menstruation we do not doubt, but that this increased fluidity was dependant upon increased fibrinousness we cannot admit. We are of opinion that the cause should be sought for in the increased absorption manifested by the blood vessel system in its partly emptied condition, by which water is abstracted from the surrounding tissues, and an attempt made by nature to preserve the normal quantity in the vessels, and thus obviate in a measure the constant drain. The blood, under these circumstances, becomes, of course, more and more watery, and tends constantly to escape by exosmose. The decided loss of fibrin appears here to have been taken for granted, as it furnished a ready and seemingly correct explanation of the etiology and pathology of the disease. Upon a careful perusal of the details we can find no evidence of an analysis having been made. On pages 26 and 28 our author, in his remarks on Chlorosis, says, "if the emmenagogue treatment should result in establishing the menstrual flow, the general health suffers just in proportion to the loss of blood sustained." "The great remedy for chlorosis is iron, in some or other of its various preparations." This treatment we know from positive experience to be highly beneficial. But Herberger, who has examined the blood of many chlorotic cases shows conclusively that, under the use of chalybeates, while the red corpuscles increase in quantity, the fibrin diminishes—the latter being converted into the former. Thus, in one case—a chlorotic female of twenty years—after the administration of iron for several weeks, the red globules had augmented from 38,060 to 98,319, while the fibrin had diminished from 3,609—the healthy average—to 1,950. This treatment—so eminently judicious—appears to us to be in direct opposition to the reasoning advanced on page 11.

Did our limits permit, we might allude to several other instances of what we are constrained to consider as defective physiology.

In conclusion, we are free to confess that we have been both pleased and instructed by the perusal of the "Obstetric Clinique;" and very confidently recommend it, therefore, to the younger portion of the profession, as well as to the great student-class of our Union—for whom it appears more especially to have been designed—as an excellent repertorium of clinical medicine.

